# A life in death: reflections of Peter

**DOI:** 10.1080/16549716.2021.2056377

**Published:** 2022-04-04

**Authors:** Tedros Adhanom Ghebreyesus, Wendy J. Graham

**Affiliations:** Director General, World Health Organization, Geneva, Switzerland; Department of Infectious Disease Epidemiology, London School of Hygiene and Tropical Medicine, London, UK

The counting of deaths has a long and notorious history. And today in the face of a global pandemic, counting continues to have enormous cultural, religious, political and economic significance. Reports of the number of COVID-19 deaths have become a daily public health message around the world, whilst debates continue to rage over definitions and modelled projections of the ‘true number’[1]. Deaths due directly or indirectly to COVID-19 are having huge effects on families, communities, health workers, society and the entire ‘industry’ of health care, services and research. Indeed, the very ‘worth’ of death is subject to investigation, as in the Lancet Commission on the Value of Death, and calls made to redress the imbalances in what are being called ‘death systems,’ which determine how death, dying, and bereavement are understood, experienced, and managed [2]. And on 16 August 2020, in the midst of this heightened attention to death, one of the champions for capturing information about mortality, tragically and unexpectedly died – Professor Peter Byass. This gentle giant was our friend, colleague and mentor, and our personal words of tribute are given in the panels below. He shared this special position in the lives of so many others in the global health community, and particularly those striving to improve the availability, quality and use of empirical data on mortality – as Peter did for over three decades and through over 300 journal articles. This Special Issue of *Global Health Action* is not only dedicated to the career – a ‘life in death’ – of this great man, but also sets out to demonstrate through eight original, commissioned articles, a key principle of all data science: to make a difference This principle lay at the very core of Peter’s work and ethic. Indeed, reflecting now on his contribution as a data scientist reveals a simple, logical pathway that seems to have operated across his entire career. We illuminate this here to introduce the eight papers comprising the Special Issue.

**Table:** 

**A personal tribute from Tedros Adhanom Ghebreyesus** The Peter Byass I knew wore many hats but he did so comfortably, elegantly and, above all, humbly. First and foremost, he was a leader and a giant in the field of global health, committed to championing science and research in this domain. What drove his research was the need to achieve impact and improvement in the health and lives of all people. His goal was to respond to real problems, by collecting insights from all people on the broader determinants of their health, in order to find real, lasting and equitable solutions. Second, Peter was a teacher and a guide, responsible for imparting great knowledge, and offering supportive counsel, to so many students and peers alike. This came from his belief that we need to build capacity, especially, in the developing world, throughout which he trained many masters and PhD students, including me. Peter’s legacy lives on through the guidance he provided to an untold number of professionals from around the world to carry forward this vital area of work and learning.

**Figure 1.**
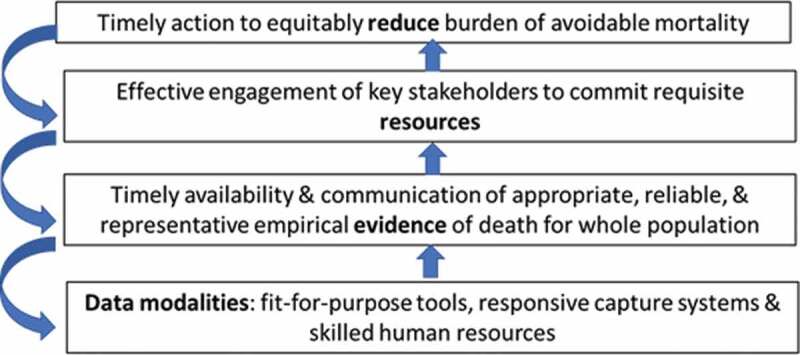
Simplified pathway showing the power & purpose of counting deaths.

The four steps in the logical pathway implicit in Peter’s contributions over three decades are shown schematically in Figure 1. This sequence is of course a simplification of reality: the process is neither unidirectional nor conducted in a vacuum. Many other drivers and influences impact at each step and operate outside of the sphere of data science, such as political instability limiting stakeholder commitment or economic shocks affecting funding for new research tools. But the simple pathway does help to highlight Peter’s ability and commitment to work at all levels – never losing sight of data mechanisms as a means to an important end – reducing burden and achieving health. This underlying logic can also be seen in much of the work described in the eight papers and which strongly reflect Peter’s legacy. Each of the papers underscore the focus of myriad bodies, including the World Health Organization, to advocate, through initiatives like the SCORE for Health Data Technical Package [3], for investment in all countries towards well-functioning civil registration and vital statistics systems that register all births and deaths, issue birth and death certificates, and compile and disseminate vital statistics, including cause of death information. Collecting good quality primary data remains a huge challenge – the WHO SCORE assessment shows that almost 40% of the world’s deaths are still not formally registered.

**Table:** 

Third, on a deeply personal level, he was a true friend, someone who was always there for me, first as a collaborator in Ethiopia, where we worked on a joint project between the Tigray Health Bureau and Mekelle University, dealing with the interplay between agriculture and health. This subject went onto become the focus of my PhD studies that Peter guided me through at Nottingham University. I also had the chance to learn from Peter’s excellence in the field of epidemiology at his beloved Umeå University. We spent countless hours discussing many subjects on all manner of areas, from the pursuit of data science to its application in Africa for the betterment of people’s health and well-being. Peter possessed so many wonderful traits, a collection which I have rarely found associated with any one person. He was very helpful, honest, friendly, warm, calm, candid, intelligent, humble, and, above all, kind. I had the great privilege to experience each of these sides of Peter, and know that many others around the world enjoyed the same opportunity. For this reason, his mission to help all, from those behind the data to those to whom he could impart his knowledge, has helped make the world a fairer, healthier place. He continues to be sorely missed.

In Papers 1 & 2, led respectively by Kobus Herbst [4] and Chodziwadziwa Kabudula [5], the value of covering whole populations through health and demographic surveillance sites (HDSS) for understanding mortality transitions and the implications for health system responsiveness are clearly illustrated. The history of HDSS, as described in Paper 1, is closely intertwined with Peter’s own career and his long-term support for the INDEPTH collaborative network, with his first paper on this topic published in 2000 for what became the Butajira HDSS in Ethiopia [7]. The challenges of multi-country and multi-site partnership are highlighted in Paper 1, and counterbalanced by demonstrating the benefits of big data and meta-analysis, which are possible by using standardized tools for data capture, quality assessment, management, and analysis. One of the most important insights achieved from these continuous, longitudinal resources – individually and collectively – arises from having sufficient size to support disaggregation of data and so to illuminate inequities in access to care and in mortality burden. The potential to miss marginalized and underserved population groups through reliance on particular sources, such as health facility-based reporting, has long provided a strong rationale for population-based systems, such as HDSS. These systems thus operate out of a strong sense of justice for making deaths count [8].

**Table:** 

***A personal tribute from Wendy Graham*** I first knew of Peter in 1988 whilst we were both working in The Gambia with the UK Medical Research Council. Our “encounter” was virtual – we never actually met physically, and indeed I only quite recently realized this was the first time our paths crossed. The circumstances illustrate so well a number of defining features of Peter. I was in The Gambia to field test a new approach to estimating levels of maternal mortality – The Sisterhood Method [6]. The Gambia was a unique place to trial this as it supported a number of the earliest health and demographic surveillance sites (HDSS) in LMICs. Little did I know at the time that it was in fact Peter who enabled this unique experiment to take place. Working as a computer scientist at the MRC, Peter had encouraged many HDSS’s in The Gambia to computerize and link their records. And in so doing, Peter demonstrated the potential of such sites for a huge variety of health and demographic research topics, some of which we see heralded today among the eight papers in this volume. This to me is a classic illustration of Peter’s visionary capacity, his ability to innovate, his commitment to local empirical data, and his modest and gentle determination to make a difference. And the topic of maternal mortality became the basis for our research collaboration and friendship which spanned over 30 years, many field trips, innumerable committees & conferences, and a multitude of journal articles. To learn just before his untimely death that he was also a Methodist Minister – dealing with death, dying and bereavement with the integrity and sincerity he also applied to his academic life, came as a surprise at the time. I now realize this was an entirely consistent role for him and one which confirms the “measure of the man” – dear Peter.

Kobus Herbst and colleagues show clearly the growing recognition over time in the complementarity of population- and health service-based information, and how linkage of these two systems often provides the most valuable evidence for stakeholders – be these patients or resource decision-makers. A comprehensive example of this is seen in Paper 2 by Chodziwadziwa Kabudula and colleagues. Here we read the story of a rural population in transition over a quarter of a century as told through the Agincourt HDSS. The challenge of an emerging communicable disease – HIV/AIDS – to an already fragile health system is documented, along with the transition to a high burden from noncommunicable diseases (NCDs), such as cardiovascular disease. The history and development of this HDSS is closely intertwined with Peter’s career [9]. He spent several months there every year and played a major part in strengthening local capacity to design, gather, analyze and use mortality and morbidity data – believing passionately in the importance of local creation and ownership of evidence. Indeed, as Kobus Herbst and colleagues note in their paper, both individual HDSS and networks such as INDEPTH, continue to be hugely important for strengthening country capacity in data science and in the other steps in Figure 1 mentioned earlier. Similarly, Paper 2 emphasizes another opportunity provided by HDSS and this is to facilitate the development, testing and refinement of new tools for capturing all aspects of the mortality burden. These advances include crucial research on the definition and capture of causes of death, as introduced in Papers 3 (Daniel Chandromohan et al. [10]) and 4 (Lucia D’Ambruoso et al. [11]).

Whilst death as an event is indisputable, determining the causes – in all senses of the word – has been controversial from medical, legal, ethical, sociological, cultural and religious perspectives for centuries. The emergence of new diseases or risk factors often re-opens debates on classification schema, as for example, in the current case for COVID-19 where essentially a time of death definition is widely used – ‘death with 28 days of a positive COVID test’, so similar to that used for maternal death – death within 42 days of the end of pregnancy, as discussed later in Paper 8 [12]. The proximate and distal causes of death are of course fundamental evidence for designing and implementing preventive and curative interventions and services. In parts of the world where medical certification of death is lacking, creative ways to capture lay reports of signs, symptoms and care-seeking prior to death have been developed over the last 70 years. Referred to as Verbal Autopsy (VA), Paper 3 describes both the pioneering work of WHO in developing standards and Peter’s contribution to this, including the computer-based algorithm he developed with colleagues back in 2003, called Inter VA [13]. The culmination of WHO’s work will lead to the release of the 2022 VA Toolkit which enables these developments to be fully accessible to LMICs and also supports capacity strengthening in their use. A development highlighted in Paper 3, and one particularly consistent with Peter’s commitment, is the adaptation and integration of tools initially devised for research purposes, such as InterVA, into routine data capture systems – so facilitating the availability of evidence on causes of death for decision-making, and thus for mortality reduction initiatives.

Causes of death termed and classified using medically-based frameworks, such as the long-established International Classification of Diseases and Related Health Problems (ICD) dating back to the 18th century, are necessary but not sufficient alone for identifying and implementing actions at scale which are equitable, appropriate, affordable and high-quality. A wide variety of systems and tools have developed over the last 50 years to look at the social determinants of specific medical causes of death, as well as the immediate circumstances surrounding the event – particularly in relation to health care. This is reflected well in the latest version of the ICD – ICD-11 which was launched by WHO in February 2022 and expanded to include extension codes for quality and patient safety factors, and traditional medicine [14]. Whilst Paper 3 discusses the WHO VA toolkit and the reporting of avoidable factors, another development is the Circumstances of Mortality Categories (COMCATs) system, as described in Paper 4. Here the authors report on a large-scale assessment of the value of additional insights provided by COMCATs for local action. In terms of almost 120,000 deaths from an HDSS in South Africa, the key circumstances contributing to fatal outcomes were limitations in the referral chain, in the quality of care, in the access to relevant interventions, in the recognition of severity, and in emergency responsiveness. As Lucia D’Ambruoso and her colleagues, conclude stakeholder engagement was enhanced not only by co-creation of the COMCATs system but also the explicit intention to meet their needs for evidence in order to act. These needs evolve over time as does the constituency of relevant stake-holders, and this is seen clearly in Papers 5 [15] and 6 [16] which both look at emerging global public health topics and ones to which Peter also devoted attention – climate change, and the Zika virus [17,18].

This Special Issue is launched in early April 2022,as is World Health Day – this year with a focus on the theme of planetary health. Described as a solutions-oriented, transdisciplinary field and social movement, planetary health analyses and addresses the impact of human disruptions to Earth’s natural systems on human health and all life on Earth [19]. Climate change is one the main examples of such disruption, and in Paper 5, Maria Nilsson and colleagues highlight the links to inequities in terms of the disproportionate adverse impact on LMICs and on marginalized population groups. The value of HDSSs is once again demonstrated in terms of revealing exposure to increased heat, drought or extreme weather events and the effects on health across long periods of time and for whole populations, so revealing disparities. The three case-studies presented from HDSSs in the INDEPTH network provide the type of rich, context-specific detail that drove Peter’s commitment [20] to locally-generated and used data, and also show the potential of these surveillance sites to prospectively capture climate change effects on health – individually and collectively. Indeed, planetary health as a scientific subject and a social movement requires massive collaboration across disciplinary and national boundaries to safeguard human survival, which INDEPTH has long-demonstrated, as described by Kobus Herbst and colleagues in Paper 1. The benefits of such large-scale collaboration are also seen clearly in Paper 6 which explores an emerging infectious disease linked with environmental change impacting on vectors – the Zika virus. Annelies Wilder Smith and thirty co-authors describe an impressive partnership across Latin America and Europe to create the ZikaPLAN research consortium to address key knowledge gaps and to strengthen research capacity to respond to other emerging diseases. A range of sustainable modalities to support the next generation of researchers in infectious neurological diseases is described in the paper, including a large suite of online training modules to enable global outreach and reduce learning inequalities – a mission to which Peter was committed and active throughout his career [21].

The last two papers in the Special Issue (Andrew Seal et al. [22] and Clara Calvert et al. [12]) appropriately focus-in on the huge global test faced over the last two years – COVID-19. The estimated global number of deaths among individuals infected with SARS-CoV-2 in the previous 28 days, by mid-February 2022, ranged hugely, from around 5.8 million [23] to 19.3 million [24]. The figures will remain estimates and the true number of deaths will never be known, as for a previous pandemic – the 1918–20 flu, where estimates range from 50 million to 100 million deaths [25]. The sudden emergence of the SARS-CoV-2 virus in late 2019 immediately presented measurement challenges, with WHO at the forefront of grappling with diagnostics, classification, and surveillance, to inform mitigation and management responses. These challenges were layered onto weak existing information systems in LMICs and led to the development of rapid mortality surveillance (RMS) tools, to which Peter contributed [26]. In Paper 7, Andrew Seal and colleagues show the importance of innovation in data capture, particularly in fragile contexts and populations – in this case, economically-vulnerable households in receipt of cash-transfers in Somalia. Using mobile phones and an adapted verbal autopsy tool employing a syndromic case definition of COVID-19, five rounds of interviews were conducted with households from June 2020 to April 2021. This example of a timely and responsive approach to an immediate need for evidence by stakeholders (here NGO actors) in order to act fits well with the logical pathway shown earlier in Figure 1. The paper also demonstrates the importance of reasonable compromise – of balancing the need for speed in getting into the field to fill a data void with the inevitable limitations of rapid surveillance. Such a compromise was acknowledged also in Peter’s work, where he repeatedly emphasized the need for continuous improvement of tools, and cautious interpretation and triangulation of findings with other sources [27].

The final paper in the Special Issue by Clara Calvert and colleagues brings together a longstanding and a new global health problem – maternal mortality and COVID-19 [12]. It is fitting for these topics to feature in the concluding article as they also represent, respectively, old and new focal areas of Peter’s career: his first paper on maternal mortality was published in 1990 [28] and, as noted earlier, three decades later he contributed to the WHO RMS for COVID-19 in 2020 [26]. Paper 8 presents the findings of a rapid systematic review of the impact of COVID-19 on populations levels of maternal mortality. Just seven studies were able to meet the inclusion criteria of the review, with five of these being representative of maternal deaths among the general population of pregnant or recently delivered women rather than just those using maternity services. Although the direction of effect across all studies was an increase in maternal mortality compared with pre-pandemic levels, the authors urge caution against over-interpretation owing to the poor quality of some studies and to only two showing a statistically significant increase. In conducting the review, the authors highlight issues of generic relevance to mortality reporting during the pandemic, including misclassification of causes, overreliance on models and projections, and the real risks of neglecting disadvantaged sub-groups of pregnant women by only using health services data – especially given robust evidence of dramatic declines in the uptake of maternity care across many countries [29]. Paper 8 provides yet another example of the underlying logic which drove so much of Peter’s work – of needing fit-for-purpose tools and approaches (here a systematic review) to generate timely, robust evidence to inform stakeholders to act (in this case to implement a service safety net for pregnant women).

**Table:** 

**Key messages from Special issue** The counting of deaths is always a means to an end and not the end itself: counting must have a chain in place to influence and inform **action**. The modalities of capturing deaths can aggravate **inequalities** in burden and access to care by excluding marginalised sub-groups. Whole population reporting, such as through HDSSs, is essential to revealing such disparities. Modelled and projected estimates of mortality will continue to be needed but they must not deter from strengthening locally-owned reporting systems generating local **empirical data.** Medically-defined **causes of death** are necessary but not sufficient alone for informing preventative and treatment actions at a population level: lay reporting, as through Verbal Autopsy tools, and the social, cultural, political, and environmental circumstances surrounding deaths, as captured by COMCAT for example, are also needed. **Innovations** in the capture, management, processing and use of mortality data, such mobile-phone based reporting, are crucial responses to constantly changing contexts, owing to climate change, or emerging diseases, such as ZIKA or COVID-19. **Capacity strengthening** of the human resources and systems for creating timely, appropriate, high quality, equitable data on deaths is a continuous and universal need. In particular it is essential that civil registration and vital statistics (CRVS) systems are improved around the world.

And so to end this Special Issue with the characteristic optimism of our dear friend and colleague – Peter, and as seen in one of his last publications [30]. Our final text panel summarizes six key messages emerging from the papers which signal priorities for delivering data for action to reduce the burden of avoidable mortality. Professor Peter Byass leaves behind a strong legacy for realizing this potential – everywhere.
